# Impact of RAAS blockers on serum potassium and mortality in a large dialysis cohort: a longitudinal analysis

**DOI:** 10.1093/ckj/sfag025

**Published:** 2026-03-03

**Authors:** Vincenzo Calabrese, Giovanni Luigi Tripepi, Domenico Santoro, Valeria Cernaro, Sabrina Mezzatesta, Francesco Mattace-Raso, Claudia Torino

**Affiliations:** Department of Internal Medicine, Erasmus MC, University Medical Center Rotterdam, Rotterdam, The Netherlands; Department of Medicine and Surgery, University of Enna “Kore”, Enna, Italy; National Research Council – Institute of Clinical Physiology, Reggio Calabria, Italy; Unit of Nephrology and Dialysis, Department of Clinical and Experimental Medicine, A.O.U. “G. Martino”, University of Messina, Messina, Italy; Unit of Nephrology and Dialysis, Department of Clinical and Experimental Medicine, A.O.U. “G. Martino”, University of Messina, Messina, Italy; National Research Council – Institute of Clinical Physiology, Reggio Calabria, Italy; Department of Internal Medicine, Erasmus MC, University Medical Center Rotterdam, Rotterdam, The Netherlands; Department of Internal Medicine, Erasmus MC, University Medical Center Rotterdam, Rotterdam, The Netherlands; National Research Council – Institute of Clinical Physiology, Reggio Calabria, Italy

**Keywords:** dialysis, longitudinal analysis, mortality, RAASIs, serum potassium

## Abstract

**Background:**

Renin–angiotensin–aldosterone system inhibitors (RAASIs) are widely used antihypertensive drugs. Due to the hyperkalaemic effect, previous 2012 Kidney Disease: Improving Global Outcomes (KDIGO) guidelines discouraged RAASI use in patients with severe chronic kidney disease (CKD). However, due to the recently discovered cardioprotective and nephroprotective effects, the 2022 KDIGO guidelines suggest their use in CKD stage 4–5. To our knowledge, few studies have explored the use of RAASIs in dialysis patients. This study aims to evaluate the impact of RAASIs on kalaemia and on mortality in a large sample of dialysis patients.

**Methods:**

We included 4764 dialysis patients from the Sicilian Registry of Nephrology, Dialysis and Transplantation. The longitudinal association between RAASI intake and serum potassium was analysed by univariate and multivariate linear mixed models. The survival models were computed through univariate and multivariate Cox models and Cox models with mixed effects.

**Results:**

The study included 4764 patients, of whom 1207 (25%) were treated with RAASis. Multivariate longitudinal models showed a direct association between RAASI intake and serum potassium {adjusted β = 0.10 [95% confidence interval (CI) 0.05–0.15], *P* < .001}. However, multivariate Cox analysis did not show any association between RAASI intake and mortality [adjusted hazard ratio 0.76 (95% CI 0.43–1.30), *P* = .31]. No differences in the impact of RAASIs on mortality were found in the analysis stratified for potassium levels (cut-off 5.1 mmol/l).

**Conclusions:**

In the present study we found, in a large cohort of dialysis patients, an independent, direct association between RAASIs and serum potassium. However, no association was found between RAASI intake and mortality. Although specific randomized controlled trials are needed to confirm our findings, RAASI intake did not seem to have a negative impact on patients’ survival, thus suggesting a re-evaluation of RAASI use in this population.

KEY LEARNING POINTS **What was known:**

- The use renin–angiotensin–aldosterone system inhibitors (RAASIs) in chronic kidney disease (CKD) patients has been controversial, due to the hyperkalaemic impact of these drugs.
- Thanks to the availability of more efficient, potassium binders, the new Kidney Disease: Improving Global Outcomes guidelines suggest their use in CKD patients with an estimated glomerular filtration rate >10 ml/min.

**This study adds:**

- The present study aims to investigate the impact of RAASIs on serum potassium and mortality in a large prospective, longitudinal cohort including only dialysis patients.

**Potential impact:**

- RAASIs increase serum potassium levels, with a difference of 0.1 mmol/l found between users and non-users. However, as no impact of RAASIs on mortality has been demonstrated, this effect is not clinically relevant. These results pave the way for the use of RAASIs in the dialysis population.

## INTRODUCTION

Renin–angiotensin–aldosterone system inhibitors (RAASIs) are widely used antihypertensive drugs. Their mechanisms of action consist of a reduction in the angiotensin-converting enzyme (ACE) effect, with a consequent reduction of angiotensin II; a competing antagonism for the alpha 1 subunit of angiotensin II receptor blockers (ARBs), which in turn lessens the sodium-related pathway of peripheral vasoconstriction; and the antagonism of aldosterone receptors, which impair the vasoconstriction and sodium retention effect of aldosterone (aldosterone antagonists). Taken together, these mechanisms reduce the vasoconstrictive effect of angiotensin and aldosterone, causing vasodilatation and, in turn, a consequent reduction in blood pressure [1]. However, as aldosterone is responsible for sodium reabsorption in the distal tubule and collecting duct through hydrogen and potassium ion excretion, impairment of the renin–angiotensin–aldosterone pathway leads to an increase in potassium levels [2]. Patients affected by chronic kidney disease (CKD) have an intrinsic risk of developing hyperkalaemia. For this reason, and due to their hyperkalaemic effect, the 2012 Kidney Disease: Improving Global Outcomes (KDIGO) guidelines [3] advised not to prescribe RAASIs in patients with an estimated glomerular filtration rate (eGFR) <30 ml/min/1.73 m^2^. In spite of these recommendations, recent studies have suggested a potential cardioprotective and nephroprotective action [4, 5] of RAASIs, also showing an increased risk for mortality and morbidity [6–8] in patients who stopped this treatment. This effect was observed in patients with and without CKD, and it is likely to be due to their impact in fibrogenesis reduction. These results, along with the availability of new, more efficient potassium binders, led the new KDIGO guidelines to allow their use in CKD patients. More specifically, according to the new guidelines, some RAASIs can be administered in patients with an eGFR >10 ml/min [9].

In contrast with CKD patients, there is not similar strong evidence that recommends the use of these drugs in dialysis patients. So far, only a few small studies have been published in peritoneal dialysis patients, aiming to evaluate the impact of RAASIs on residual renal function [10–13].

In haemodialysis (HD), two studies evaluated the impact of RAASIs on cardiovascular mortality [14, 15]. However, no longitudinal data were available in both studies.

This study aims at longitudinally evaluating the impact of RAASIs on serum potassium and on all-cause mortality in a large sample of dialysis patients from the Sicilian Registry of Nephrology, Dialysis and Transplantation (SRNDT).

## MATERIALS AND METHODS

The study conforms with the guidelines of the Italian Data Protection Authority and the Helsinki Declaration. We analysed data from the SRNDT, a collection of regional data instituted in 2008 by regional lows. The SRNDT was established with the aim of collecting data on CKD patients for scientific purposes (decree 03423/08). Informed consent is requested from all patients whose data are entered in the registry. However, as specified in article 1, no formal approval from ethical committees is needed to analyse data, as it is made available only in anonymous form.

Demographic and clinical characteristics, dialysis details, comorbidities and mortality of patients undergoing chronic renal replacement therapy, as well as information regarding renal transplantation in Sicily, were collected on the REGDIAL web platform (Cooperativa EDP La Traccia, Matera, Italy). Data were extracted from the SRNDT in accordance with ethical standards and respecting privacy.

### Study population and laboratory data

In this prospective cohort study, we included HD patients entered in the SRNDT from 1 January 2018 to 31 December 2020. Among a total of 6451 patients, 1220 were excluded because of a lack of potassium measurement and 467 because RAASI information was missing. Thus 4764 patients (for a total of 56 964 measures) were included in the statistical analyses.

Patients had been on regular HD for a median follow-up of 38 months [interquartile range (IQR) 13–83], with a median total of 10 visits (IQR 4–28), and were being treated with standard bicarbonate dialysis with non-cellulosic membrane filters of various types (enaxone, elixone, polyethersulfon, polyacrylonitrile, polyamide, polymix, polyethene-polyvinyl alcohol, polymethylmethacrylate). A total of 3400 patients were treated with various antihypertensive drugs (1050 on monotherapy with ACE inhibitors, calcium channel blockers, α- and β blockers, vasodilators, diuretics or other drugs; 1153 on double therapy; 822 on triple therapy; and 383 patients on quadruple or quintuple therapy with various combinations of these drugs).

Among 4764, 1207 were treated with RAASIs. RAASI and serum potassium measurements were collected for each patient since the start of dialysis treatment. Only patients with no missing values for these variables were included.

The main demographic, somatometric, clinical and biochemical characteristics of the study population are detailed for intake of RAASIs and death (Table 1 and Supplementary Table 1, respectively).

**Table 1:** Clinical, demographic and laboratory characteristics of the study population and divided by RAASi intake.

Variables	Non-treated (*n* = 3557)	Treated (*n* = 1207)	*P*-value	Correlation
Age (years), mean ± SD	66.8 ± 14.5	64.5 ± 14.6	.001	−0.071
Dialytic age (years), median (IQR)	0 (0–1)	0 (0–1)	.71	0.003
Male, *n* (%)	2178 (61)	782 (65)	.028	0.032
Potassium (mmol/l), mean ± SD	4.9 ± 0.8	5.0 ± 0.8	.001	0.048
Albumin (g/dl), mean ± SD	3,60 ± 0,50	3,64 ± 0,51	.057	0.029
CRP (mg/dl), median (IQR)	3.1 (1.1–7.3)	2.9 (1.0–6.1)	.041	−0.041
Phosphate (mg/dl), mean ± SD	4.7 ± 1.3	4.8 ± 1.3	.010	0.038
TSAT (%), mean ± SD	21.6 ± 12.5	22.7 ± 12.7	.017	0.039
Iron (ng/ml), *n* (%)	1665 (47)	641 (53)	.001	0.055
Diuresis (once a day), *n* (%)	1183 (33)	580 (48)	.001	0.133
Cholesterol (mg/dl), mean ± SD	158 ± 42	157 ± 42	.620	−0.008
Use of ASA, *n* (%)	1447 (41)	709 (59)	.001	0.158
Use of calcium channel antagonists, *n* (%)	1236 (35)	697 (58)	.001	0.204
Use of folic acid, *n* (%)	512 (14)	247 (20)	.001	0.072
Use of vitamin B12, *n* (%)	158 (4)	86 (7)	.001	0.053
Use of β-blocker, *n* (%)	1170 (33)	554 (46)	.001	0.118
Use of immunosuppressants, *n* (%)	8 (0)	7 (1)	.057	0.028
Arrythmia (yes), *n* (%)	139 (9)	44 (6)	.008	−0.048
Arterial hypertension (yes), *n* (%)	1546 (69)	660 (80)	.001	0.171
Diabetes (yes), *n* (%)	705 (32)	272 (36)	.024	0.038
Heart failure (yes), *n* (%)	228 (10)	50 (7)	.003	−0.055
Chronic liver disease (yes), *n* (%)	190 (9)	39 (5)	.002	−0.056
COPD (yes), *n* (%)	228 (10)	48 (6)	.001	−0.056

Pearson’s or Spearman’s test was performed for normal or non-normal distribution, respectively.

### Data collection

Laboratory and clinical data were collected locally from the SRNDT referents as part of normal clinical practice and then entered in the platform. Laboratory data included serum phosphate, haemoglobin, C-reactive protein, iron, transferrin, ferritin, potassium, calcium, intact parathyroid hormone (PTH), albumin, glucose, triglycerides, cholesterol, bicarbonate, alkaline phosphatase, fractional urea clearance (Kt/V) and β_2_-microglobulin. All laboratory data were measured pre-dialysis with the exception of blood urea, which was measured both pre-dialysis and post-dialysis to compute Kt/V. However, only pre-dialysis blood urea was included in the analysis, whereas post-dialysis measurement was used only for Kt/V calculation. Clinical data included blood pressure levels, residual diuresis, previous comorbidities [dementia, hemiplegia, liver disease, history of arterial hypertension, vascular disease, chronic obstructive pulmonary disease (COPD), malignancy with/without metastasis, heart failure, psychiatric disease, dyslipidaemia, prostatic hypertrophy] as well as pharmacological treatment such as antihypertensives, folic acid, calcium carbonate, cholecalciferol, insulin, aspirin, allopurinol, phosphorous binder, calcium mimetics, cortisone, erythropoiesis-stimulating agents, iron supplementation, immunosuppressive treatment, proton pump inhibitors, paricalcitol and vitamin B12. Details of the registry are described elsewhere [16].

### Statistical analysis

Data were described as mean ± standard deviation (SD), median and interquartile range (IQR) or proportion, as appropriate. The distribution of variables was investigated by the Kolmogorov–Smirnov test followed by graphic evaluation.

Serum potassium, as well as the quantitative confounders, was included in the analysis as a continuous variable.

The proportion of missing data differed for each variable. In detail, Kt/V was not available in 22% of measures, body mass index in 8%, albumin in 29%, PTH in 41%, haemoglobin in 7%, serum calcium in 4%, serum phosphate in 4%, ferritin in 42%, bicarbonate in 54%, systolic pressure in 11% and CRP in 59%. Other variables included in the multivariate models had <0.01% of missing data. Missing values were related neither to the centre that provided the data nor to specific characteristics of the patients and thus were considered at random. As mixed effects models are well equipped to handle missing (at random) response data if estimated using likelihood methods, we did not impute or recover them. The longitudinal association between RAASI intake and serum potassium was analysed by univariate and multivariate linear mixed models (LMMs). In adjusted analyses, we included as potential confounders all variables related to RAASI intake and serum potassium with a *P*-value <.2 in Pearson’s or Spearman’s tests for normal or non-normal distribution, respectively (age, sex, albumin, hypertension, calcium receptor blocker, COPD, CRP, diabetes, diuresis, serum iron, heart failure, serum phosphate, transferrin saturation (TSAT), visit and Kt/V).

We tested various univariate and multivariate models that were compared according to Akaike information criterion (AIC). Models were computed as follow: one univariate random intercept LMM was performed using the identification code (ID) as a random variable; two univariate LMMs performing both random slope and random intercept in which RAASIs or visit was used as a random slope variable and ID as a random intercept variable; one multivariate random intercept model using ID as a random intercept variable; two multivariate LMMs using visit or RAASIs as a random slope variable; two multivariate LMMs computing both random slope and random intercept in which RAASIs or visit is used as a random slope variable separately and ID for the intercept and the last multivariate LMM in which both RAASIs and visit were used in the same model for the random slope and the ID for the random intercept.

Variables related to severe hyperkalaemia (serum potassium >6.5 mmol/l) in the RAASI group were tested with a generalized estimating equation model for repeated measures. All variables related to hyperkalaemia with a *P*-value <.2 were included in the multivariate model as potential confounders [albumin, diabetes, phosphate, use of calcium channel antagonists, TSAT, use of folic acid, COPD, dialytic age, acetyl salicylic acid (ASA), diuresis, CRP, sex, heart failure and ferritin].

The survival models were computed as follow: one univariate Cox model without a random effect; one univariate Cox model with mixed effects including the ID as a random effect; one multivariate Cox model with mixed effects including ID as a random effect; one multivariate Cox model with mixed effects including the interaction between RAASIs and visit among the fixed effects variables and ID as a random effect. Similar to the LMM, the AIC was applied to choose the best-fitting Cox model.

Each recording (named ‘visit’) was ordered chronologically and numbered in ascending order. This variable was treated as a discrete variable and included in the LMM and Cox models both as a fixed variable and a random variable.

A sensitivity analysis was performed dividing the cohort by serum potassium levels (cut-off 5.1 mmol/l), age (cut-off 65 years) and sex.

## RESULTS

Clinical, demographic and laboratory data of the whole study population, and divided according to RAASi use, are detailed in Table 1. The mean age was 66 ± 15 years, 62% were male, 33% were diabetic and arterial hypertension history was reported in 74% of patients. Hyperkalaemia at baseline was present in 1848 patients. Among these, 506 were in the RAASI group, corresponding to 42% of this population, a proportion significantly higher than that in the non-treated group (38%) (*P* = .01). Serum potassium was also slightly but significantly higher in patients using RAASis (5.0 ± 0.8 versus 4.9 ± 0.8; *P* = .001). In treated patients, mean age, CRP, prevalence of heart failure, COPD and arrhythmia were lower than in not-treated patients, whereas albumin and phosphate levels, prevalence of diabetes and arterial hypertension were higher (Table 1).

### Cross-sectional analysis

Pearson’s correlation analysis showed a significant relationship between RAASI use and serum potassium (*r* = 0.048, *P* < .001). The list of the correlates of potassium are summarized in Supplementary Table 2.

The correlation between RAASI use and serum potassium was confirmed in a univariate linear regression model [β = 0.09 (95% CI 0.04–0.14), *P* < .001]. This suggests that RAASI use is related to an increase in serum potassium of ≈0.1 mmol/l. However, this association was not confirmed in the multivariate analysis, adjusted for the full set of confounders, at baseline [β = 0.09 (95% CI −0.02–0.19), *P* = .10] (Supplementary Table 3).

### Longitudinal analysis

Univariate longitudinal analysis showed a direct association between RAASI use and serum potassium [β = 0.05 (95% CI 0.03–0.07), *P* < .001].

A multivariate model, adjusted for potential confounders, confirmed this association [adjusted β = 0.10 (95% CI 0.05–0.15), *P* < .001] (Table 2), with fixed effects of this model explaining 43% of the variability. This means that 43% of the potassium values were explained by the variable included in this multivariate model.

**Table 2:** LMM showing the direct association between the use of RAASIs and serum potassium.

	Longitudinal univariate random intercept analysis			Longitudinal multivariate analysis		
	β	95% CI	*P*-value	Adjusted β	95% CI	*P*-value
RAASIs (yes)	0.05	0.03–0.07	<.001	0.100	0.05–0.15	<.001
Age (years)				0.003	0.001–0.005	.001
Male				−0.011	−0.06–0.04	.646
Albumin (g/dl)				0.188	0.15–0.23	<.001
Hypertension (yes)				0.004	−0.05–0.06	.124
Use of calcium channel antagonists (yes)				0.03	−0.008–0.07	.122
COPD (yes)				−0.081	−0.154 to −0.008	.03
CRP (g/dl)				−0.002	−0.0033 to −0.0006	.014
Diabetes (yes)				−0.087	−0.14 to −0.03	.001
Diuresis (yes)				−0.116	−0.17 to −0.06	<.001
Serum iron (ng/ml)				−0.019	−0.05–0.01	.199
Heart failure (yes)				−0.061	−0.14–0.02	.151
Serum phosphate (mg/dl)				0.077	0.06–0.09	<.001
TSAT (%)				0.001	0.0003–0.0024	.011
Visit				0.011	0.008–0.015	<.001
Kt/V				0.034	−0.018–0.087	.201

β refers to the slope of the univariate LMM, whereas adjusted β refers to the slope of the multivariate LMM.

Interaction between RAASIs and visit was significant in the univariate model [β = 0.05 (95% CI 0.04–0.09), *P* = .05] but not in the multivariate model [β = 0.02 (95% CI −0.04–0.08), *P* < .53]. Thus it was not included in the analysis.

The multivariate model showed a significant association between hyperkalaemia and albumin, diabetes, phosphate, TSAT, COPD, dialytic age, ASA and diuresis (see Supplementary Table 4).

### Survival analysis

**Figure 1:**
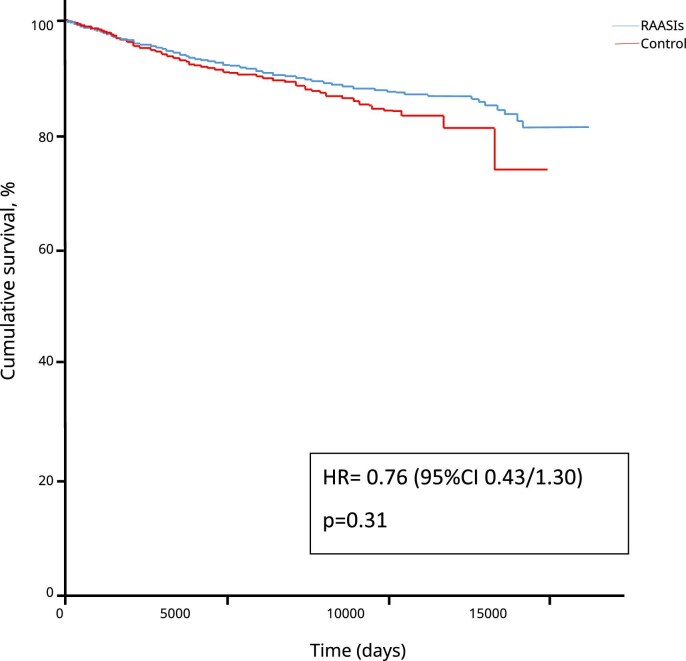
Kaplan–Meier curves showing no difference in cumulative survival due to RAASI use.

**Figure 2:**
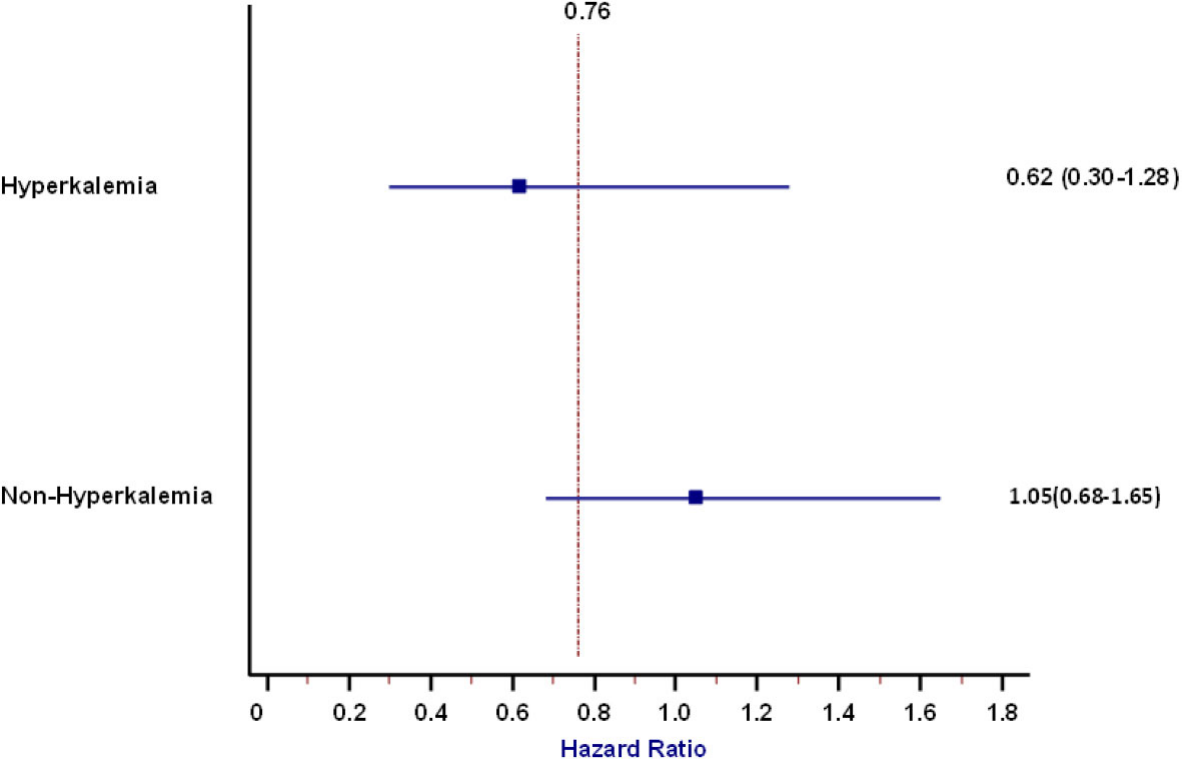
Impact of RAASIs on mortality in patients with and without hyperkalaemia (cut-off 5.1 mmol/l).

Death was registered in 1999 patients. Among these, 481 (24%) were treated with RAASIs. Univariate Cox regression analysis did not show a direct association of RAASI use and mortality [hazard ratio (HR) 1.03 (95% CI 0.93–1.15), *P* = .55]. To confirm these results, time-dependent analyses were performed and different multiple Cox models were tested and compared by using the AIC. Our analysis showed that the best multivariate model was the one including a mixed effects Cox model adjusted for potassium, serum phosphate, albumin, CRP, Kt/V, visit, age, sex, arrythmia, arterial hypertension, heart failure, COPD, diabetes, calcium channel inhibitors, folic acid and ASA. However, neither the univariate Cox model with mixed effects nor the multivariate Cox model with mixed effects showed a significant impact of RAASI use on mortality [HR 0.82 (95% CI 0.66–1.03), *P* = .09 and HR 0.76 (95% CI 0.43–1.30), *P* = .31, respectively] (Fig. 1, Table 3). In line with these results, in the analysis stratified for potassium levels, no differences in mortality rates were found in patients with and without hyperkalaemia (Fig. 2). Subgroup analysis for age and sex was performed, but no significant interaction was found (*P* = .54 and *P* = .80, respectively). Collinearity was investigated and a variance inflation factor (VIF) >5 was considered as positive for the presence of collinearity. This analysis showed no collinearity in our models (highest VIF was 1.21).

**Table 3:** Cox models showing the direct association between the use of RAASIs and mortality.

	Cross-sectional univariate analysis			Longitudinal multivariate analysis		
Variables	HR	95% CI	*P*-value	HR	95% CI	*P*-value
RAASIs (yes)	1.03	0.93–1.15	.55	0.756	0.44–1.304	.31
Serum potassium (mmol/l)				0.549	0.42–0.72	<.001
Age (years)				1.163	1.13–1.20	<.001
Male				1.359	0.72–2.60	.35
Albumin (g/dl)				0.350	0.23–0.54	<.001
Hypertension (yes)				0.598	0.29–1.26	.17
Arrhythmia (yes)				1.468	0.75–2.84	.026
Use of anticoagulant (yes)				0.975	0.60–1.60	.92
Use of calcium channel antagonists (yes)				2.071	1.25–3.42	.005
COPD (yes)				2.259	1.02–5.00	.045
CRP (mg/dl)				1.012	0.998–1.026	.095
Diabetes (yes)				6.613	3.57–12.27	<.001
Use of folic acid (yes)				0.689	0.39–1.22	.2
Heart failure (yes)				7.751	3.40–17.69	<.001
Visit				0.881	0.85–0.92	<.001
Kt/V				0.223	0.12–0.45	<.001
Serum phosphate (mg/dl)				1.196	1.02–1.40	.027

Interaction was tested between RAASIs and diabetes, diuresis, heart failure, sex and Kt/V and no significant interaction was found.

## DISCUSSION

In the present study performed in a large cohort of dialysis patients, we found a direct association between RAASIs and serum potassium. Such an effect is independent of comorbidities, dialysis performance and domiciliary therapy. Despite potassium increasing in patients treated with RAASIs, no association with mortality was found. Indeed, despite our multivariate model showing a significant statistical relationship between potassium and mortality, RAASIs have no significant impact on mortality in either hyperkalaemia or in non-hyperkalaemia. In our sample, hyperkalaemia was manifest in 52% of the measurements, whereas hypokalaemia was <2%. Consequently, considering that hypokalaemia was so rarely represented, we may assume that the serum potassium HR in the multivariate analysis indicates a protective efficacy of normal serum potassium compared with high serum potassium.

The incidence of hyperkalaemia in CKD patients is ≈40–50% [17–19]. Moreover, patients in whom hyperkalaemia occurs have an increased risk of a new occurrence, with a progressively shorter time between the episodes [20]. This can be explained by both the reduced ability to eliminate acid with impaired kidney function, where kidneys tend to excrete cations and reabsorb potassium to balance the ion charges, and by the reduced nephron mass and consequent reduction of excretion activity. Furthermore, in these patients the hyperkalaemic power of RAASIs can be increased. Indeed, kalaemia tends to be increased in patients treated with RAASIs because the activity of Na/K-APTase reducing serum potassium is inhibited. Whereas compensatory mechanisms such as the aldosterone paradox or K^+^/H^+^ exchange well manage kalaemia in patients with normal renal function, in CKD patients these mechanisms are not efficient enough [21]. Accordingly, in our cohort, RAASIs are slightly related to a potassium increase, and the impact of RAASIs on kalaemia can be physiologically explained. Indeed, RAASIs-related hyperkalaemia is caused by decreased urinary potassium excretion in the distal and collecting tubules, as well as by increased movement of potassium from the intracellular and extracellular space [21]. Among antihypertensive drugs, RAASIs are able to reduce myocardial interstitial fibrosis and cardiac myocyte apoptosis, protect by cellular oxidative stress [22, 23] and impair the Klotho–fibroblast growth factor 23 axis, reducing fibroblast activation [24, 25]. Findings also suggest a potential role in reducing mortality and hospitalization [26]. Indeed, according to some observational studies, RAASI discontinuation seems to be related to an increase in all-cause mortality and the end-stage renal disease risk [27, 28] in both CKD patients and in patients without CKD [29, 30]. In light of these recommendations, RAASIs were considered recommendable enough to be added in trials including patients with CKD stage 4 [31, 32]. Indeed, the structure of these trials required the use of RAASIs as inclusion criteria with an eGFR ≥20 ml/min/1.73 m^2^.

In a large analysis of 2639 patients with CKD stage 1–5 and arterial hypertension, RAASI therapy seemed to decrease the renal function decline by ≈20%, with more beneficial effects in patients with CKD stage 3b–5 [33].

Similarly, a meta-analysis based on clinical trials showed a reduction in heart failure of 33% in patients treated with ACEs or ARBs. However, only five studies were included in this meta-analysis, with a cumulative sample of 1115 patients. Furthermore, no impact on mortality or cardiovascular mortality has been detected, in keeping with our results [34]. In contrast, no longitudinal analyses have been performed in dialysis patients. Only one open-label randomized controlled trial (RCT) compared patients who took ARBs to patients who did not take ARBs in a sample of 366 HD patients. In this RCT, no significant differences in mortality were detected, even though the authors highlighted a trend of mortality reduction in the RAASI group [15].

A large observational study was performed in dialysis patients with heart failure evaluating the efficacy of a sacubitril/valsartan association, but patients took RAASIs in both groups and they did not compare them with patients not treated with RAASIs [15].

The impact on mortality and morbidity of hyperkalaemia is well known [35, 36]. As serum potassium levels deviate from normal levels, rates of morbidity and mortality increase, independent from the comorbidities. Indeed, hyperkalaemia seems to increase mortality more than hypokalaemia, although the latter has an increased risk compared with normokalaemia [35, 36]. This increased mortality is explained by the higher risk of arrhythmia in patients with deviated serum potassium concentrations, such as in patients with rapid potassium intradialytic oscillations [37]. Furthermore, the mortality rate has been evaluated in many observational studies, and all of them showed higher mortality in hyperkalaemic patients, even though significance was not found in all studies, and the risk was analysed in differently in the various studies (OR, percentage, HR) [38].

Similar to the ALCHEMIST Trial, which showed no impact of spironolactone on potassium concentration or on cardiovascular events and mortality, our analysis showed no impact of RAASI use on the same outcomes, including mortality, despite these treatments significantly increasing serum potassium. The reason why RAASIs do not have an impact on mortality in spite of their hyperkalaemic effect can be explained by the fact that this effect was limited in size. Indeed, only a mean increase of 0.1 mmol/l has been estimated by our model, thus having no clinical impact on mortality. Moreover, in our stratified analysis, a significant impact of RAASIs on mortality was not seen in the hyperkalaemia or non-hyperkalaemia patients. This showed the independence of the impact of RAASIs on mortality by serum potassium, despite RAASIs seeming to slightly increase serum potassium.

The major strengths of our study are the large sample size and the longitudinal evaluation. Moreover, as reported in the methods section, we used many different statistical models and reported the best method according to the AIC. All methods showed equivalent results in terms of impact on mortality, and this improved the likelihood of our results and forced the internal validity of our analysis. RAASI status was monitored longitudinally throughout the study and changes were registered in 980 patients. This enhanced the internal validity of our analysis.

One important limitation is the lack of mortality causes. Based on the unknown cardiovascular events of these patients, we cannot analyse if their impact on mortality due to hyperkalaemia is balanced by their cardioprotective effects, as our analysis was limited to all-cause mortality. Furthermore, our study included only patients from one Italian region with a high prevalence of Caucasians, and this has an evident impact on its generalizability. Finally, no information about the type of RAASis taken is available, thus no comparison between different treatments can be done. Another limitation was the lack of information about the use of potassium-sparing agents and the RAASI doses.

In conclusion, the present study detected no association between RAASI use and mortality even though an independent, direct association between RAASIs and serum potassium was found. These results suggest considering several factors other than kalaemia before discontinuing RAASI treatment, in line with current management strategies for advanced conservative CKD. Additionally, new potassium binders may help manage serum potassium levels in dialysis patients when hyperkalaemia occurs. Although specific RCTs are needed to confirm our findings, this longitudinal analysis performed in a large cohort of dialysis patients is able to suggest that RAASI use did not seem to have a negative impact on patients’ survival, thus suggesting a re-evaluation of RAASI use in this population.

## Supplementary Material

sfag025_Supplemental_File

## Data Availability

The data were retrieved from the SRNDT (http://www.crtsicilia.it/PUBLIC/RegistroRSNDT/CentriDialisiETx.aspx).
