# Supplementary material for: Impact of RAAS blockers on serum potassium and mortality in a large dialysis cohort: a longitudinal analysis
Source: Clin Kidney J. 2026 Mar 3;19(4):sfag025. doi: 10.1093/ckj/sfag025 (PMC13042221; doi:10.1093/ckj/sfag025)
Supplement: sfag025_Supplemental_File [file sfag025_supplemental_file.docx]

**Supplementary**

**Supplementary Table 1** Main demographic, clinical and laboratory characteristics in the cohort divided according to the vital status.

| Variable | Alive (n=2765) | Death (n=1999) | p | Correlation |
| --- | --- | --- | --- | --- |
| RAASIs, yes | 726 (26) | 481 (24) | 0.086 | -0.025 |
| KT/V | 1,35±0,30 | 1,32±0,30 | 0.016 | -0.043 |
| BMI, kg/m2 | 26,03±5,14 | 25,66±5,08 | 0.026 | -0.037 |
| Albumin, g/dl | 3,67±0,48 | 3,53±0,52 | 0.001 | -0.138 |
| HDL, mg/dl | 42,41±12,20 | 41,44±12,01 | 0.053 | -0.040 |
| Triglyceride, mg/dl | 125 [88-174] | 121 [88-164] | 0.071 | -0.031 |
| Hemoglobin, g/dl | 10,56±1,31 | 10,43±1,35 | 0.001 | -0.044 |
| Calcium, mg/dl | 8,77±0,83 | 8,69±0,85 | 0.001 | -0.045 |
| Ferritin, ng/ml | 147 [60-317] | 180 [74-389] | 0.001 | 0.079 |
| CRP, mg/dl | 3.0 [1.0-5.6] | 3.4 [1.1-8.3] | 0.001 | 0.080 |
| Phosphate, mg/dl | 4,83±1,32 | 4,63±1,36 | 0.001 | -0.074 |
| Age, year | 62,07±14,90 | 72,02±11,94 | 0.001 | 0.337 |
| Use of ASA | 1198 (43) | 958 (48) | 0.002 | 0.046 |
| Use of Calcium antagonist | 1159(42) | 774(39) | 0.027 | -0.032 |
| Use of Folic acid | 474 (17) | 285 (14) | 0.007 | -0.039 |
| Arrythmia, yes | 116 (7) | 124 (10) | 0.001 | 0.063 |
| Arterial hypertension, yes | 1345 (76) | 861 (70) | 0.001 | -0.072 |
| Diabetes, yes | 472 (27) | 505 (41) | 0.001 | 0.149 |
| Heart failure, yes | 101 (6) | 177 (14) | 0.001 | 0.146 |
| COPD, yes | 117 (7) | 159 (13) | 0.001 | 0.107 |

**Supplementary Table 2** correlation among variables and potassium

| Variables | r | p | Variables | r | p |
| --- | --- | --- | --- | --- | --- |
| Use of folic acid | 0.023 | 0.406 | Ferritin, ng/ml | 0.066 | 0.001 |
| Albumin, g/dl | 0.149 | 0.001 | Use of Iron | 0.035 | 0.016 |
| Use of Platelets Antiaggregant | 0.011 | 0.441 | Serum phosphate, mg/dl | 0.166 | 0.001 |
| Arrhythmia | -0.036 | 0.046 | Use of Immunosuppressors | -0.014 | 0.341 |
| Use of Beta blockers | -0.003 | 0.824 | Hypertension, yes | 0.005 | 0.776 |
| BMI | -0.027 | 0.084 | KTV | 0.061 | 0.001 |
| Calcium, mg/dl | 0.042 | 0.005 | COPD, yes | -0.094 | 0.001 |
| Use of Calcium antagonist | -0.028 | 0.05 | CRP, mg/dl | -0.105 | 0.001 |
| Cholesterol | 0.003 | 0.877 | iPTH | 0.026 | 0.112 |
| Death, yes | 0.003 | 0.81 | Use of RAASIs | 0.048 | 0.001 |
| Diabetes, yes | -0.069 | 0.001 | heart failure, yes | -0.084 | 0.001 |
| Use of Diuretics | -0.115 | 0.001 | triglycerides | 0.006 | 0.724 |
| Hemoglobin, g/dl | 0.108 | 0.001 | TSAT, % | 0.082 | 0.001 |
| Liver chronic diseases, yes | 0.072 | 0.001 | Use of Vitamin B12 | 0.029 | 0.044 |
| Age, year | -0.042 | 0.004 |  |  |  |

**Supplementary Table 3** Multivariate baseline regression analysis showing the direct association between use of RAASIs and Serum potassium.

|  | Cross sectional univariate linear regression analysis | | | Cross sectional multivariate linear regression analysis | | |
| --- | --- | --- | --- | --- | --- | --- |
|  | Coeff | 95% CI | p | Coeff | 95% CI | p |
| RAASIs, yes | 0.09 | 0.04/0.14 | <0.001 | 0,087 | -0,52/0,69 | 0,108 |
| Age, year |  |  |  | 0,004 | -0,10/0,11 | 0,019 |
| Sex, male |  |  |  | 0,053 | 0,05/0,06 | 0,286 |
| Albumin, g/dl |  |  |  | 0,129 | 0,03/0,22 | 0,026 |
| Hypertension, yes |  |  |  | 0,045 | -0,07/0,16 | 0,425 |
| Use of Calcio ant |  |  |  | 0,014 | -0,10/0,12 | 0,776 |
| COPD, yes |  |  |  | -0,164 | -0,26/-0,07 | 0,036 |
| CRP, mg/dl |  |  |  | -0,003 | -0,16/0,15 | 0,178 |
| Diabetes, yes |  |  |  | -0,088 | -0,09/-0,08 | 0,085 |
| Diuresis, yes |  |  |  | -0,150 | -0,25/-0,05 | 0,003 |
| Serum Iron, ng/ml |  |  |  | 0,083 | -0,015/0,180 | 0,089 |
| heart failure, yes |  |  |  | -0,070 | -0,24/0,10 | 0,409 |
| serum phosphate, mg/dl |  |  |  | 0,094 | 0,06/0,13 | <0,001 |
| Tsat, % |  |  |  | 0,009 | 0,006/0,013 | <0,001 |
| KT/V |  |  |  | -0,083 | -0,25/0,08 | 0,329 |

**Supplementary Table 4** Univariate and Multivariate GEE analysis for repeated measures showing the OR of hyperkalemia in the RAASIs subsample.

| Variable | Univariate | | | Multivariate | | |
| --- | --- | --- | --- | --- | --- | --- |
|  | OR | 95% CI | p-value | OR | 95% CI | p-value |
| Age, year | 0.999 | 0.992-1.006 | 0.843 | - | - | - |
| Dialytic age, year | 1.025 | 1.010-1.039 | 0.001 | 1.020 | 1.004-1.035 | 0.012 |
| Sex, male | 0.869 | 0.716-1.053 | 0.152 | 0.887 | 0.721-1.092 | 0.260 |
| Albumin, g/dl | 1.365 | 1.163-1.602 | <0.001 | 1.250 | 1.076-1.453 | 0.004 |
| CRP, mg/dl | 0.997 | 0.992-1.001 | 0.115 | 0.998 | 0.993-1.470 | 0.384 |
| Phosphate, mg/dl | 1.139 | 1.065-1.218 | <0.001 | 1.135 | 1.065-1.210 | <0.001 |
| TSAT, % | 1.006 | 1.002-1.011 | 0.003 | 1.005 | 1.001-1.009 | 0.036 |
| Ferritin, ng/ml*100 | 1.026 | 1003-1.050 | 0.026 | 1.019 | 0.995-1.044 | 0.094 |
| Diuresis, l/die | 0.715 | 0.570-0.898 | 0.004 | 0.706 | 0.537-0.928 | 0.012 |
| Cholesterol, mg/dl | 0.999 | 0.998-1.001 | 0.215 | - | - | - |
| Use of ASA | 1.392 | 1.066-1.818 | 0.151 | 1.393 | 1.104-1.757 | 0.005 |
| Use of Calcium channel antagonists | 1.246 | 0.918-1.689 | 0.158 | 1.292 | 0.988-1.689 | 0.061 |
| Use of Folic acid | 1.356 | 1.102-1.666 | 0.004 | 1.207 | 0.969-1.504 | 0.094 |
| Use of Vit B12 | 1.107 | 0.807-1.517 | 0.529 | - | - | - |
| Use of Β-blocker | 1.159 | 0.832-1.613 | 0.381 | - | - | - |
| Use of Immunosuppressors | 0.529 | 0.051-5.505 | 0.595 | - | - | - |
| Arrythmia, yes | 0.980 | 0.738-1.301 | 0.889 | - | - | - |
| Arterial hypertension, yes | 0.911 | 0.786-1.057 | 0.220 | - | - | - |
| Diabetes, yes | 0.686 | 0.532-0.884 | 0.004 | 0.075 | 0.567-0.991 | 0.036 |
| Heart failure, yes | 0.627 | 0.346-1.138 | 0.125 | 0.750 | 0.383-1.470 | 0.402 |
| Liver chronic disease, yes | 0.987 | 0.615-1.583 | 0.956 | - | - | - |
| COPD, yes | 0.502 | 0.322-0.784 | 0.002 | 0.589 | 0.361-0.960 | 0.034 |
